# Human papillomavirus (HPV) vaccination uptake (initiation and completion): factors associated with uptake of the human papillomavirus (HPV) vaccine among young people living with HIV in Uganda

**DOI:** 10.1186/s12913-026-15035-7

**Published:** 2026-06-27

**Authors:** Scovia Nalugo Mbalinda, Aggrey Semeere, Derrick Amooti Lusota, Jean Hunleth, Michelle Silver, Dan Kabonge Kaye, Miriam Nakalembe

**Affiliations:** 1 Department of Nursing, School of Health Science, College of Health Sciences, Makerere University, Kampala, Uganda; 2 Infectious Disease Institute, Kampala, Uganda; 3 Department of Surgery, School of Medicine, Washington University at St Louis, St Louis, MO USA; 4 Department of Obstetrics and Gynecology, School of Medicine, College of Health Sciences, Makerere University, Kampala, Uganda; 5 Department of Nursing, School of Health Sciences, Makerere University, P.O Box 7072, Kampala, Uganda

**Keywords:** HPV, HPV vaccination, Adolescents, HIV-positive, Young people

## Abstract

**Introduction:**

The prevalence of human papillomavirus (HPV) infection and related illnesses is disproportionately high in women and young people living with HIV. Vaccination against high-risk HPV types has the potential to reduce HPV- associated disease burden dramatically. However, HPV vaccine uptake has been variable and suboptimal in most countries, including Uganda, with low levels of both initiation and completion of the HPV doses. The purpose of this research was to identify determinants of HPV vaccine uptake and completion among young girls living with HIV.

**Methods:**

A cross-sectional study was conducted among 274 adolescents and young people living with HIV who were selected from an antiretroviral therapy (ART) clinic in a district Hospital ART clinic, Uganda. Data were collected between February and June 2024. The data were collected using a structured questionnaire examining HPV vaccine uptake (initiation and completion). Bivariate and multivariate analyses were conducted to assess factors associated with HPV vaccine uptake using SPSS version 21 software.

**Results:**

This study involved 274 participants, with a mean age of 20.3 years ranging from 15-24 years. The proportion who initiated HPV vaccination in this study was 68.2% (187/274), and HPV vaccine dose completion (2 doses and more) was 24.1% (66/274). The uptake of HPV vaccination was significantly associated with age 15 to 19 years (AOR 2.9, 95% CI 1.352-6.1 *P* = 0.006), having been taught about HPV vaccine and cervical cancer at school (AOR 8.7 95% CI 2.8–24.7, *P* = < 0.001), living within a distance of less than 5 km from the health facility (AOR 5.9, 95% CI 2.2–16.1, *P* = < 0.001), and availability of HPV vaccines at the health facility (AOR 5.2, 95% CI 1.9–14.5, *P* = < 0.001). The completion of HPV vaccination was significantly associated with age 20 to 24 years (AOR 1.9, 95% CI 1.057–3.499, *P* = 0.032).

**Conclusion:**

The initiation and completion of HPV vaccination among HIV- positive girls are both crucial for preventing HPV related diseases and cancers. In this study, the completion rate is very low. Vaccination against human papillomavirus (HPV) must be prioritised in the HIV-positive youth population. The findings suggest a need for targeted policies to improve vaccine uptake among HIV-positive youth, including better education, healthcare provider engagement, and system support for follow-up care. This will guide targeted interventions in achieving the WHO’s target of 90% immunising all adolescent girls by 2030.

**Supplementary Information:**

The online version contains supplementary material available at https://doi.org/10.1186/s12913-026-15035-7.

## Introduction

Globally, cervical cancer, a malignancy caused by the human papillomavirus(HPV), ranks fourth in terms of frequency among women [1]. In 2022, globally there were to be an estimated 660,000 new cases and 350,000 fatal cases [1]. Low- and middle-income countries (LMICs) have the highest incidence and mortality rates from cervical cancer [2]. With an age-standardized incidence rate of 47.5 per 100,000 and a mortality rate of 25 per 100,000, Uganda has some of the highest rates of cervical cancer worldwide in terms of both incidence and mortality [3, 4].

The burden of HPV infections and cervical cancer worldwide adds to the impact that HIV has on women, especially young women and adolescent girls from LMICs [5]. A comprehensive analysis of the HPV prevalence in sub-Saharan Africa revealed that women living with HIV (WLWH) had a much higher HPV prevalence of 54% and a higher rate of co-infections [6]. HIV-positive women had a greater prevalence of any HPV infection or multiple HPV infections (53.6/22.6%) compared to HIV-negative women (26.5/7.3%) [7]. HIV-positive individuals are more likely than HIV-negative individuals to get HPV infections, diseases, and malignancies associated with HPV [8–11].

The HPV vaccine is highly effective at preventing HPV infections that can lead to cervical cancer and other HPV-related cancers [12]. The HPV vaccine effectively prevents cervical, anal, oropharyngeal, penile, vulvar, and vaginal cancer infections [13, 14], providing strong protection for at least 10–11 years [15]. World Health Organization (WHO) recommends at least two doses of HPV vaccination (with a minimum 6-month gap) and, if feasible, three doses should be administered to people known to be immunocompromised or HIV-positive (independent of age or use of antiretroviral medication) [16]. The best time to deliver the HPV vaccine is prior to the start of sexual activity. Research shows that many teenagers start having sex by the time they are 14 years old; thus getting vaccinated early is essential to maximizing protection against HPV [17–19]. Immune responses to vaccination in HIV-positive individuals are frequently suboptimal. However, although they can improve with antiretroviral therapy (ART), they often stay lower and diminish more quickly than in HIV-negative individuals [8–11].

In Uganda, HPV vaccination was launched in 2015, and the 2-dose HPV vaccine series has since been integrated into the Uganda National Expanded Program on Immunisation [17] (UNEPI) by adopting two strategies, the school-based approach and the static (with selected outreach) routine immunisation services and focusing on young girls aged 9–14 [20]. However, current WHO guidelines have acknowledged the effectiveness of administering a single dose of HPV vaccine and have advised revising the dosage schedules to provide countries with the option to provide either one or two doses to girls aged 9–14 years, and at least two doses or more for immunocompromised individuals, including adolescent girls living with HIV [21]. Uganda is yet to adopt these strategies.

In Uganda, the uptake of HPV vaccines is still below average, with notable differences observed between various areas and demographic groups. Low HPV vaccination uptake among school girls in Lira City, Uganda, at 19.3%, is linked to school education, clinic exposure, and health worker recommendations [22, 23]. A survey of young women in Lake Victoria fishing communities revealed low HPV vaccine uptake, attributed to factors like limited awareness, frequent mobility, and school absenteeism [23]. A 2016 Uganda Demographic and Health Survey revealed that 78% of adolescent girls aged 10–14 had not received HPV vaccination, with factors like school attendance, age, ethnicity, and socioeconomic status influencing vaccination uptake [24]. There is limited information available about the uptake and completion of the HPV vaccine, as well as associated factors, among young female people living with HIV.

## Theoretical framework

Several theoretical frameworks guided the study of factors influencing HPV vaccination uptake in Uganda. The Health Belief Model (HBM), focusing on perceived vulnerability, severity, advantages, obstacles, action cues, and self-efficacy, provided a robust framework for understanding and addressing these factors [25]. The HBM provided a framework for examining the beliefs and perceptions influencing individuals’ decisions to receive the HPV vaccine. The Risk Information Seeking and Processing for Prevention (RISP) model offered a comprehensive understanding of how individuals process information about HPV vaccination, considering factors such as risk perceptions, information sufficiency, and social norms [26]. This model helped understand the factors influencing vaccination intentions and behaviours. Andersen’s Behavioral Model, a well-established model in the field, further enhanced the scientific rigour of the research, providing a comprehensive understanding of the behavioural factors influencing HPV vaccination uptake [27].

## Materials and methods

### Study design and setting

This was a cross-sectional study of adolescents and young girls living with HIV attending the Mityana General Hospital ART clinic. Data were collected from February 2024 to June 30, 2024.

### Study site

The study was conducted at Mityana General Hospital. Mityana General Hospital is located in the town of Mityana, Mityana District, in the Central Region of Uganda, about 85 km (53 mi) East of Mubende Regional Referral Hospital, the regional referral hospital, and approximately 69 km (43 mi) west of Mulago National Referral Hospital, the largest hospital in the country.

### Study population

Youngs females living with HIV were selected from the adolescent ART clinic through a consecutive sampling procedure. At the site, the research assistants recruited all the available participants in the ART clinic. They enrolled those who fulfilled the inclusion criteria (aged 15 to 24 years, provided informed consent or assent, not sick to consent and exclusion criteria not present at the ART clinic at the time of data collection until the sample size was obtained.

### Sample size determination

We used the Kish Leslie sample size determination formula, assuming a prevalence of 22% [21] and a margin of error of 5% at a 95% confidence interval. The sample size was 255 respondents. On adjusting for non-response, at a rate of 10%, the final calculated sample size was 280 respondents. However, during data collection, we were able to enroll 272 (97.2%) respondents.

### Variables and measurements

As a dependent variable, HPV vaccine uptake (i.e., initiating and completing the vaccine) was measured by having a vaccination card that shows the number of doses received and the memory of getting an injection on the left upper arm if the child was 9–15 years old.

Initiation was defined as receiving at least one of the recommended two- or more-dose series of the HPV vaccine, and completion was defined as receiving or completing two or more doses of the vaccine.

Independent variables in this study included socio-demographic factors (e.g., age, religion, tribe, main caregiver education level, and employment status), health system factors (e.g., availability of vaccines, accessibility to health facilities, etc.), and knowledge, attitudes, and information about HPV vaccination.

Data were collected through face-to-face interviewer-administered questionnaires in a private room. This questionnaire was developed from the literature and adopted for this study. The data collected from participants included socio-demographic characteristics (age, religion, tribe, main caregiver, education level and status, employment status of caregiver), knowledge about HPV vaccination, attitudes towards HPV vaccination, information on HPV vaccination, uptake and factors affecting the uptake of HPV vaccination among adolescents.

### Data management and analysis

The data was collected using Kobo Collect v2023.2.4 and later exported into Excel 2016. The Excel file was exported to SPSS version 21 software for analysis. The mean, median, frequency tables, and standard deviations of continuous variables were derived at univariate analysis.

The proportion of those who initiated and completed HPV vaccination was computed as the number of participants with evidence of vaccination cards indicating the number of HPV vaccines received over the total participant population. To assess factors associated with the initiation and completion of the HPV vaccine, we utilized Pearson’s chi-square test for categorical explanatory factors, and the student t-test for continuous explanatory variables.

The variables that were found statistically significant in bivariate analysis with a p-value of less or equal to 0.05 were considered for multivariate logistic regression. Analyses were done using SPSS version 21. Stepwise logistic regression models were built to identify independent predictors for initiating and completing the HPV vaccine. During model development, all predictor variables with a p-value of ≤ 0.2 at bivariate analysis were considered for inclusion in the multivariate logistic regression model. Collinearity was assessed using a correlation matrix and cross-checked using the variance inflation factor set at 10. In case two variables were associated (*P* < 0.05), the variable explaining the largest variability (smaller P-value at univariate analysis) was retained. Significance was set at 0.05, and all of the analyses were two-tailed.

### Quality assurance

The research team was trained, and research tools were pre-tested among facilities not included in the study; this provided clarity, relevance, and comprehension of the tool and experienced research assistants were used as part of the quality assurance methods. Field supervision initiatives ensured the collection of high-quality data through coordinating the activities of their team, reporting progress, and addressing any issues that may arise in a timely manner daily supervision and spot checks by field supervisors can help identify and rectify any inconsistencies or incomplete data at the time of collection.

The research team was the only one with access to the participants’ data, which was securely stored. The data collection tool was translated to the local language (Luganda). to ensure enhanced data quality, reduced data loss, inclusivity by reaching non-native speakers, and equity in research by ensuring all segments can participate. This approach respects cultural diversity, builds trust, and results in honest responses, providing reliable data collection.

#### Ethics approval and consent to participate

Ethical reviews and approval were obtained from the Research Ethics Committee of the School of Health Sciences, College of Health Sciences at Makerere University (#MAKSHSREC-2023-611) and the Uganda National Council for Science and Technology (HS3685ES). Administrative clearance and permissions were also obtained from the management of Mityana General Hospital. Written informed consent was obtained from young people 18 years and above. For adolescents below 18 years, consent from the parents/guardians and subsequently assent from the adolescents was obtained. Participation was voluntary, and all the interviews were conducted in private settings to ensure the confidentiality of the participants, and participation was voluntary.

## Results

### Socio‑demographic characteristics of the participants

A total of 274 (97.2%) participants were enrolled between February and June. The mean age of participants was 20.32 years (SD = 2.8), with an age range of 15–24 years. Most participants, 123 (44.9%), were Catholics by religion and were from the Baganda tribe, 217 (79.2%). Of the participants, 169 (61.7%) lived with their parents, 164 (59.9%) were currently out of school, and 110 (40.1%) were still in school. When asked about their level of education, 163 (59.5%) had secondary education, 81 (29.6%) had completed primary education, 11 (4.0) had completed tertiary education, and 19 (6.9%) had no education. 247 (90.1%) of the participants had employed caregivers, and 192 (70.1%) resided in urban settings. See Table 1 below.

**Table 1:** Socio-demographic characteristics of the participants

Variable	Frequency (*n* = 274)	Percentage (%)
**Age (Mean 20.32, standard deviation 2.815**		
Religion Catholic Anglican Born again Muslim	123 65 59 27	44.9 23.7 21.5 9.9
**Tribe**		
Muganda Other	217 57	79.2 20.8
**With whom are you staying with?**		
With parents Others	169 105	61.7 38.3
**Family type at household level**		
Nuclear family Extended family	187 87	68.2 31.8
**Current education status**		
At school Out of school	110 164	40.1 59.9
**level of education finished**		
No education Primary education Secondary education Tertiary education	19 81 163 11	6.9 29.6 59.5 4.0
**Residence**		
Urban Rural	192 82	70.1 29.9
**Employment status of caretaker**		
Employed Unemployed	247 27	90.1 9.9

### Uptake and completion of the HPV vaccine among female adolescents living with HIV/AIDS attending the ART clinic

The HPV vaccine was administered to more than half of the participants, 187 (68.2%) of whom 121 (64.7%) had received one dose, 42 (22.5%) had received two doses, and 24 (12.8%) had received three doses. When asked about where they received the HPV vaccines from, most of the participants reported schools 157 (84.0%) for the first dose, 53 (80.3%) for the second dose, and 20 (83.3%) for the third dose. The proportion of HPV vaccine uptake in this study was 68.2% (187/274), and HPV vaccine dose completion was 24.1% (66/274). See Table 2 below.

**Table 2:** Knowledge, information, and uptake of HPV vaccine among female adolescents living with HIV/AIDS attending a district Hospital ART clinic

Variable	Frequency (*n*)	Percentage (%)
**Knowledge about HPV vaccine**		
Ever heard of HPV Vaccine (*n* = 274)		
Yes No	182 92	66.4 33.6
HPV vaccine is used to prevent Human papillomavirus infection and cervical cancer (*n* = 182)		
Yes No I don’t know	149 8 25	81.9 4.4 13.7
Recommended dose of HPV vaccine (*n* = 182)		
One dos Two doses I don’t know	89 49 44	48.9 26.9 24.2
The ideal time HPV vaccine best recommended (*n* = 182)		
Less than 9 years 9–14 years Can be provided at any age I don’t know	19 66 53 44	10.4 36.3 29.1 24.2
**Information about HPV vaccine**		
Ever received information about HPV vaccine.		
Yes NO	138 136	50.4 49.6
Ever been taught about HPV or cervical cancer at school		
Yes No	119 155	43.4 56.6
Availability of awareness creation about cervical cancer or HPV vaccine at school or community		
Yes No	147 127	53.6 46.4
Need more information about HPV Vaccination.		
Yes No	261 13	95.3 4.7
**Uptake and completion of HPV vaccination**		
Have you received the HPV Vaccine? (*n* = 274)		
Yes No	187 87	68.2 31.8
If YES, how many doses? (*n* = 187)		
1 dose 2 doses 3 doses	121 42 24	64.7 22.5 12.8
HPV vaccine completion (*n* = 274)		
Yes No	66 208	24.1 75.9
HPV vaccine completion among those vaccinated (*n* = 121)		
Yes No	66 55	54.5 45.5

### Knowledge, attitudes, and information about HPV vaccine among female adolescents attending district Hospital ART clinic

Table 2 above shows that most participants, 182 (66.4%), had heard of the HPV vaccine, and of those, 149 (81.9%) knew that the HPV vaccine is used to prevent HPV infection and cervical cancer. When asked about the recommended doses of the HPV vaccine, 49 (26.9%) identified that the recommended doses of the HPV vaccine, i.e. two or more doses, 89 (48.9%) one dose, and 44 (24.4%) didn’t know. While 66 (36.3%) knew the ideal age for HPV vaccine was between 9 and 14 years, 53 (29.1%) said that it could be provided at any age; about half 138 (50.4%) had received information about the HPV vaccine, and 119 (43.4%) had been taught about HPV or cervical cancer at school, 147 (53.6%), reported the availability of HPV awareness creation at school or in the community, and 261 (95.3%), agreed that they needed more information about the HPV vaccine.

**Fig. 1:**
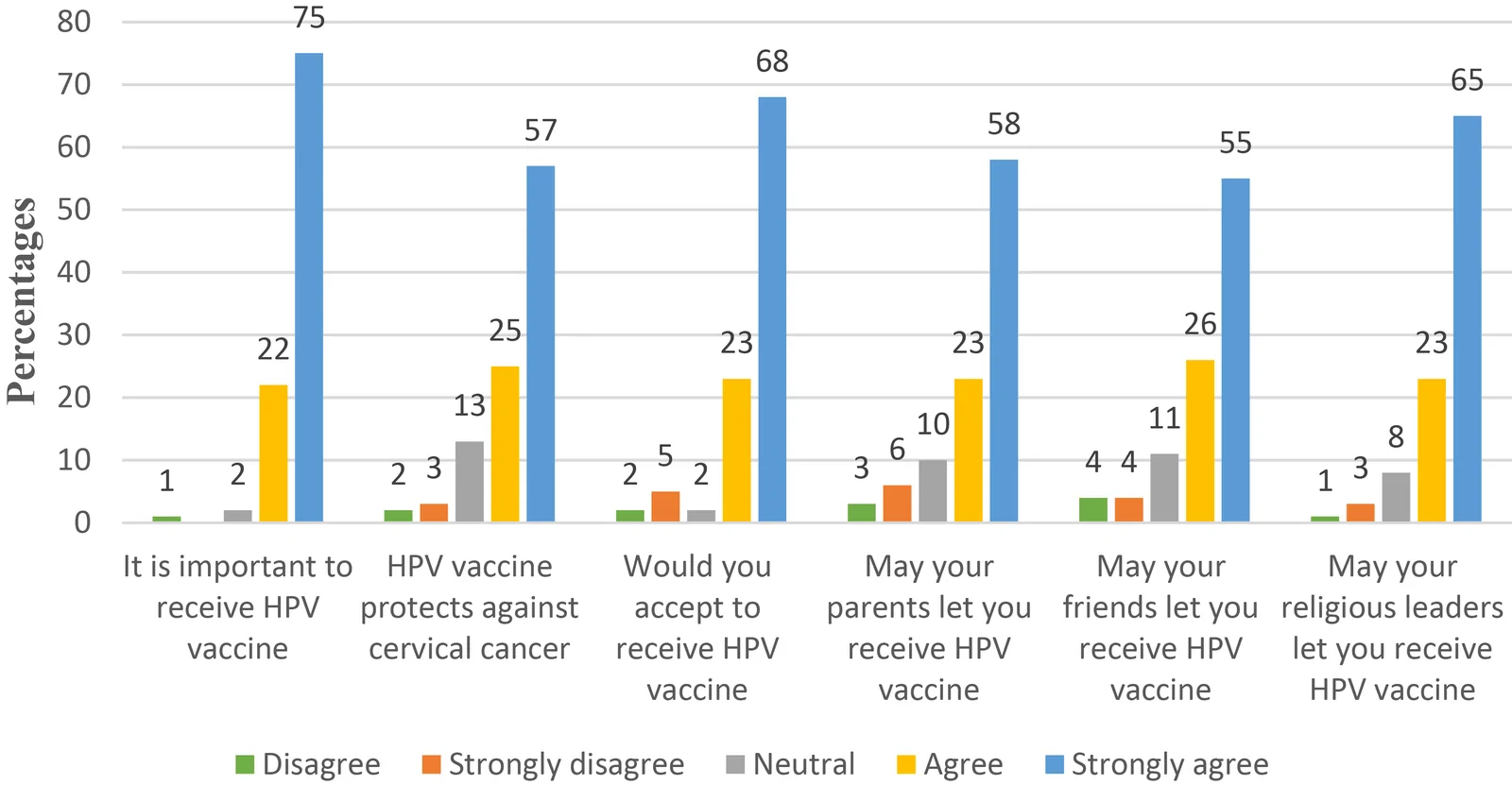
Attitudes towards HPV vaccination

Figure 1 shows the attitudes of the respondents about the HPV vaccine. Most participants, 205 (74.8%), strongly agreed that it is important to receive the HPV vaccine, 62 (22.6%) agreed, and 3 (1.1%) disagreed. When asked whether they would accept to receive the HPV vaccine, 188 (68.6%) strongly agreed, 62 (22.6%) agreed, and 14 (5.1%) disagreed. Most participants (58.4%, *n* = 160) strongly agreed that their parents would permit them to receive the HPV vaccine, while 23.0% (*n* = 63) agreed. Additionally, 9.9% (*n* = 27) were neutral on the matter, 2.9% (*n* = 8) disagreed, and 5.8% (*n* = 16) strongly disagreed.In addition, 152 (55.5%) strongly agreed that their friends convinced them to receive the HPV vaccine, 70 (25.5%) agreed, 31 (11.3%) were neutral, 11 (4.0%) disagreed, and 10 (3.6%) strongly disagreed.

**Fig. 2:**
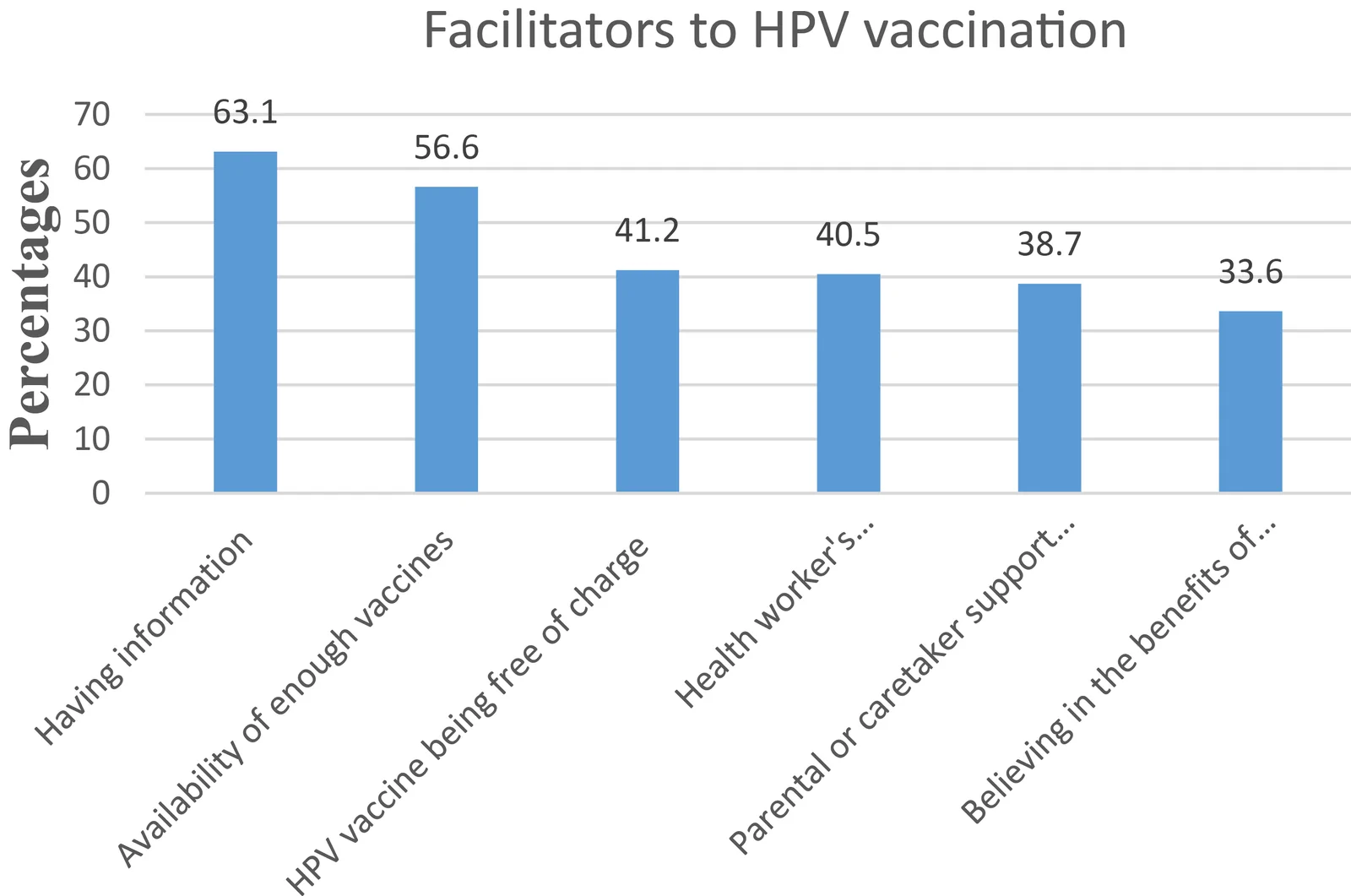
Facilitators to uptake of HPV vaccination

Figure 2 shows the facilitators of HPV vaccination. Participants mentioned the main facilitators were having information 173 (63.1%), having enough vaccines 155 (56.6%), HPV vaccines being free of charge 113 (41.2%), recommendations from health workers 111(40.5%), and Parental or caretaker support toward HPV vaccination 106 (38.7%).

**Fig. 3:**
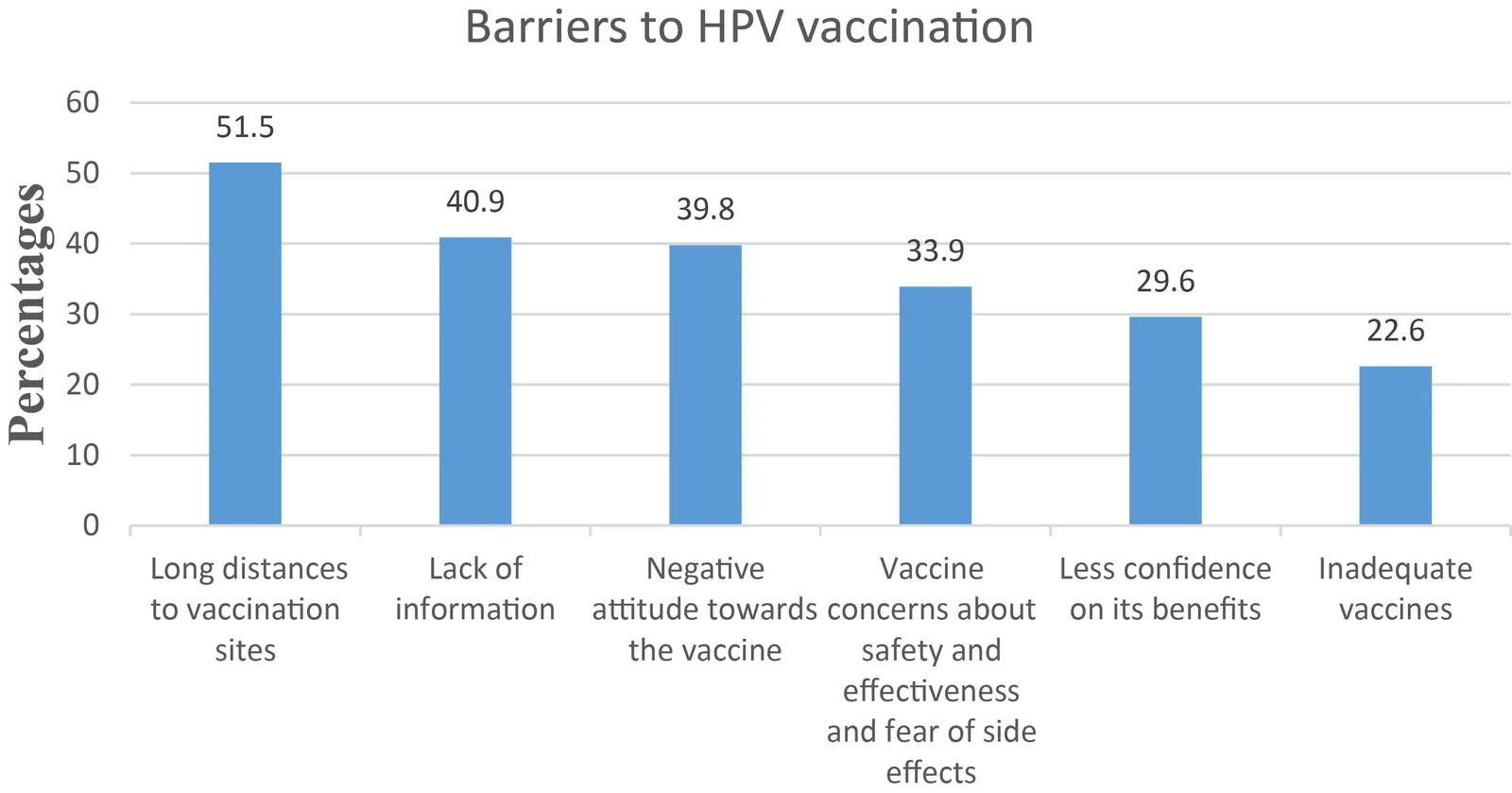
Barriers to uptake of HPV vaccination

Figure 3 shows the barriers to HPV vaccination. Long distances to the vaccination sites 141 (51.5%), lack of knowledge and information about the HPV vaccine 112 (40.9%), negative attitudes towards the vaccine 109 (39.8%), Vaccine concerns about safety and effectiveness, misconceptions, and fear of side effects 93 (33.9%) were some of the barriers to receiving the HPV vaccine reported by the participants.

### Bivariate and multivariate regression results of factors associated with uptake of HPV vaccination among adolescents attending District Hospital ART clinic

Table 3 shows the bivariate and multivariate analyses of factors associated with HPV vaccine uptake. The following were significantly associated with HPV uptake: Aadolescents who received the HPV vaccine were more likely to reside in urban centres (cOR 2.16, 95% CI 1.26–3.72, *P* = 0.005), to have ever heard of HPV vaccine (cOR 4.32, 95% CI 2.51–7.44, *P* = < 0.001), to have received information about HPV vaccine (cOR 5.73, 95% CI 3.20-10.24, *P* = < 0.01), or to have been taught about HPV vaccine and cervical cancer at school (cOR 10.80, 95% CI 5.33–22.11, *P* = < 0.001). Availability of awareness creation and campaigns about HPV vaccine (cOR 3.24, 95% CI 2.00-5.84, *P* = < 0.001), living within a distance of less than 5 km from the health facility (cOR 5.56, 95% CI 2.47–12.50, *P* = < 0.001), recommendation from health workers (cOR 3.55, 95% CI 2.02–6.22, *P* = < 0.001), and availability of HPV vaccines (cOR 9.981, 95% CI 4.57–21.81, *P* = < 0.001) were associated with HPV vaccine uptake.

Multivariate logistic regression analysis found that uptake of HPV vaccination remained significant after controlling for respondent age 15 to 19 years (AOR 2.88, 95% CI 1.35–6.14, *P* = 0.006), taught about HPV vaccine and cervical cancer at school (AOR 8.72 95% CI 2.77–24.73, *P* = < 0.001), living within a distance of less than 5 km from the health facility (AOR 5.90, 95% CI 2.16–16.12, *P* = < 0.001), availability of HPV vaccines at the health facility (AOR 5.23, 95% CI 1.89–14.51, *P* = < 0.001) (Table 3 below).

**Table 3:** Bivariate and multivariate regression results of factors associated with uptake of HPV vaccination among adolescents attending the district Hospital ART clinic

Variable	Uptake of HPV vaccine		cOR (95% CI)	*P*-value	Adjusted OR (95% CI)	*P*-value
	YES *n*= (187) *n* (%)	NO *n*= (87) *n* (%)				
**Socio-demographic characteristics**						
**Age (in years)**						
15–19 20–24	76 (40.6) 111 (59.4)	25 (28.7) 62 (71.3)	1.70 (0.98–2.94) Reference	0.058	2.88 (1.356.14) Reference	**0.006**
**Religion**						
Catholic Anglican Born again Muslim	88 (47.1) 46 (24.6) 37 (19.8) 16 (8.6)	35 (40.2) 19 (21.8) 22 (25.3) 11 (12.6)	Reference 0.87 (0.24–1.53) 0.601 (0.27–1.53) 0.59 (0.24–1.37)	0.760 0.286 0.313		
**Tribe**						
Muganda Others	148 (79.1) 39 (20.9)	69 (79.3) 18 (20.7)	Reference 1.01 (0.54–1.89)	0.975		
**Family type**						
Nuclear family Extended family	130 (69.5) 57 (30.5)	57 (65.5) 30 (34.5)	1.20 (0.70–2.06) Reference	0.508		
**Current education status**						
At school Out of school	82 (43.9) 105 (56.1)	28 (32.2) 59 (67.8)	1.65 (0.96–2.82) Reference	0.068		
**Level of education**						
No education Primary education Secondary education Tertiary education	9 (4.8) 52 (27.8) 118 (63.1) 8 (4.3)	10 (11.5) 29 (33.3) 45 (51.7) 3 (3.4)	Reference 1.02 (0.26–4.01) 1.49 (0.37–6.05) 2.96 (0.60–14.73)	0.120 0.981 0.579		
**Residence**						
Urban Rural	141(75.4) 46(24.6)	51(58.6) 36(41.4)	2.16 (1.26–3.72) Reference	**0.005**	1.20(0.59–2.43) Reference	0.612
**Employment status**						
Employed Unemployed	172(92.0) 15(8.0)	75(86.2) 12(13.8)	1.84 (0.82–4.11) Reference	0.14		
**Knowledge**,** attitude and information about HPV vaccination**						
**Ever heard of HPV vaccine**						
Yes No	144 (77.0) 43 (23.0)	38(43.7) 49(56.3)	4.32 (2.51–7.44) Reference	**< 0.001**	1.85(0.87–3.94) Reference	0.110
**Ever received information about HPV vaccine**						
Yes No	118 (63.1) 69 (36.9)	20 (23.0) 67 (77.0)	5.74 (3.20 − 10.24) Reference	**< 0.001**	0.84 (0.32–2.25) Reference	0.733
**Taught about HPV or cervical cancer at school**						
Yes No	109 (58.3) 78 (41.7)	10 (11.5) 77 (88.5)	10.76 (5.34 − 22.11) Reference	**< 0.001**	8.72 (2.77–24.73) Reference	**< 0.001**
**Availability of awareness creation about HPV or cervical cancer at school or community**						
Yes No	118(63.1) 69(36.9)	29 (33.3) 58 (66.7)	3.40 (2.00–5.84) Reference	**< 0.001**	0.55 (0.19 1.64) Reference	0.285
**Health system factors**						
**Distance from home to the health facility**						
0–5 kms 6–10 kms More than 10 kms	130 (69.5) 45 (24.1) 12 (6.4)	37 (42.5) 31 (35.6) 19 (21.8)	5.56 (2.48–12.50) 2.42 (1.35–4.35) Reference	**< 0.001** **0.003**	5.90 (2.16–16.12) 4.16 ( 0.57 30.23) Reference	**0.001** 0.123
**Health providers talk to you about HPV or cervical cancer**						
Yes No	100(53.5) 87(46.5)	23(26.4) 64(73.6)	3.20 (1.8–5.58) Reference	**< 0.001**	0.06 (0.00-1.19) Reference	0.064
**Health providers recommend to you HPV vaccine**						
Yes No	102 (54.5) 85 (45.5)	22 (25.3) 65 (74.7)	3.55 (2.02–6.22) Reference	**< 0.001**	4.83 (0.53–43.97) Reference	0.162
**Are HPV vaccines available in nearest health facility**						
Yes No	94 (50.3) 93 (49.7)	8 (9.2) 79 (90.8)	9.98 (4.57–21.81) Reference	**< 0.001**	5.23 (1.89–14.51) Reference	**0.001**

### Bivariate and multivariate regression results of factors associated with completion of HPV vaccination among adolescents attending District Hospital ART clinic

Bivariate analyses showed that adolescents who completed the HPV vaccine were more likely to be from the Baganda tribe (cOR 2.74, 95% CI 1.46–5.12, *P* = 0.002) availability of HPV vaccines at nearby health facilities (cOR 2.20, 95% CI 1.18–4.12, *P* = < 0.014) were statistically significant.

Multivariate logistic regression analysis found that completion of HPV vaccination remained significantly associated with age 20 to 24 years (AOR 1.923, 95% CI 1.057–3.499, *P* = 0.032), and being a Muganda by tribe (AOR 2.20, 95% CI 1.13–4.28, *P* = 0.021) (Table 4 below).

**Table 4:** Bivariate and multivariate regression results of factors associated with completion of HPV vaccination among adolescents attending district Hospital ART clinic

Socio-demographic characteristic	Completion of HPV vaccine		cOR (95% CI)	*P*-value	AOR (95%CI)	*P*-value
	Not fully vaccinated *n*= (208) *n* (%)	Fully vaccinated *n*= (66) *n* (%)				
Age (in years)						
15–19	70(33.7)	31 (47.0)	Reference		Reference	
20–24	138(66.3)	35 (53.0)	1.746(0.95–3.06)	0.052	1.923(1.06–3.49)	0.032
**Religion**						
Catholic	99 (47.6)	24 (36.4)	Reference			
Anglican Born again Muslim	50 (24.0) 40 (19.2) 19 (9.1)	15 (22.7) 19 (28.8) 8 (12.1)	1.13 (0.42–3.04) 0.71 (0.26– 1.95) 0.58 (0.23–1.47)	0.811 0.51 0.249		
**Tribe**						
Muganda Others	174 (83.7) 34 (16.3)	43 (65.2) 23 (34.8)	2.74 (1.46–5.12) Reference	**0.002**	2.20 (1.13–4.28) References	**0.021**
**Family type**						
Nuclear family Extended family	147 (70.7) 61 (29.3)	40 (60.6) 26 (39.4)	1.57 (0.880 − 2.79) Reference	0.127	1.41(0.75–2.64) Reference	0.284
**Current education status**						
At school Out of school	81 (38.9) 127 (61.1)	29 (43.9) 37 (56.1)	0.81 (0.46–1.43) Reference	0.471		
**Level of education**						
No education Primary education Secondary education Tertiary education	16 (7.7) 62 (29.8) 123 (59.1) 7 (3.4)	3 (4.6) 19 (28.8) 40 (60.6) 4 (6.1)	Reference 0.56 (0.16–2.04) 0.54(0.142 − 2.03) 0.33 (0.06 − 1.87)	0.388 0.359 0.210		
**Residence**						
Urban Rural	150(72.1) 58(27.9)	42(63.6) 24(36.4)	1.48 (0.82– 2.65) Reference	0.191	1.12(0.59-2.)0 Reference	0.724
**Employment status**						
Employed Unemployed	187 (89.9) 21 (10.1)	60 (90.9) 6 (9.1)	0.890 (0.343– 2.309) Reference	0.811		
**Ever heard of the HPV vaccine?**						
Yes No	140 (67.3) 68 (32.7)	42 (63.6) 24 (36.4)	1.18 (0.65– 2.10) Reference	0.582		
**Have you ever received information about the HPV vaccine?**						
Yes No	107 (51.4) 101 (48.6)	31 (47.0) 35 (53.0)	1.19 (0.69 − 2.08) Reference	0.527		
**Availability of awareness creation about HPV or cervical cancer at school or community**						
Yes No	113(54.3) 95 (45.7)	34 (51.5) 32 (48.5)	1.120 (0.643–1.949) Reference	0.690		
**Distance from home to the health facility**						
0–5 kms 6–10 kms More than 10 kms	126 (60.6) 59 (28.4) 23 (11.1)	41 (62.1) 17 (25.8) 8 (12.1)	1.07 (0.44–2.57) 0.89 (0.46–1.69) Reference	0.882 0.711		
**Health providers talk to you about HPV or cervical cancer**						
Yes No	97(46.6) 111(53.4)	27(40.9) 39(59.1)	1.26 (0.72–2.21) Reference	0.416		
**Health providers provide you with information about HPV or cervical cancer**						
Yes No	96(46.2) 112(53.8)	27(40.9) 39(59.1)	1.238 (0.706–2.170) Reference	0.456		
**Health providers recommend to you HPV vaccine**						
Yes No	96 (46.2) 112 (53.8)	28 (42.4) 38 (57.6)	1.16 (0.66–2.03) Reference	0.596		
**Are HPV vaccines available in nearest health facility**						
Yes No	86 (41.3) 122 (58.7)	16 (24.2) 50 (75.8)	2.20 (1.18–4.12) Reference	**0.014**	1.70(0.86–3.36) Reference	0.127

## Discussion

In this study of adolescent girls and young women living with HIV attending an ART clinic in Uganda, we found that while HPV vaccine initiation was relatively high at 68.2%, completion of the recommended two or more doses was notably low at 24.1%. Initiation of HPV vaccination was significantly associated with being aged 15–19 years, receiving education about HPV and cervical cancer at school, living within 5 km of the health facility, and the availability of the vaccine at the clinic. In contrast, completion of the vaccine series was more likely among participants aged 20–24 years.

HIV-positive girls require HPV vaccination due to the increased risk of contracting the virus and decreased risk of eliminating the viral infection, thus increasing the risk of developing HPV-related illnesses and malignancies. Despite antiretroviral therapy, HIV-positive individuals’ immune responses may be suboptimal, making HPV vaccination crucial for protecting them against HPV-related risks. Therefore, this study aimed to assess the factors associated with HPV vaccination uptake and completion among HIV-positive young girls.

The initiation and completion of HPV vaccination among HIV-positive girls are both crucial for preventing HPV-related diseases and cervical cancer. In this study, about 68% initiated HPV vaccination, but the completion rate was very low (24%). This is low compared to the World Health Organisation (WHO), which set goal to eradicate cervical cancer by 2030, aiming to have 90% of girls fully vaccinated with the HPV vaccine by age 15 [28]. This is very low compared to a study conducted in the United States, which reported completion rates of about 60% [29]. Reduced protection against HPV infection is one consequence of poor completion rates; this, in turn, can raise the risk of HPV-related illnesses like cervical cancer [13]. Because of their increased vulnerability to HPV-related health consequences, high-risk populations, such as HIV-positive girls, should prioritise HPV vaccination as a preventive intervention. The optimal protection against HPV-related malignancies and illnesses is ensured by completing the HPV vaccine series. To improve the chances of preventing cervical cancer and other HPV-related illnesses among HIV-positive girls, interventions should be implemented to strengthen social mobilisation, outreach, vaccination strategies, and education to increase vaccine uptake.

Knowledge of HPV is crucial in determining the uptake and completion of the vaccine [30]. Low knowledge about HPV vaccination, particularly among adolescents, can lead to misconceptions and hinder its effectiveness, affecting the uptake and utilisation of vaccination programs [31, 32]. In this study, we found that 66.4% of participants knew about the HPV vaccine; 81.9% knew it prevented infection and cervical cancer. However, only 26.9% knew the recommended doses, and 36.3% knew the ideal age for the vaccine (9–14 years). Furthermore, adolescents who heard about the HPV vaccine were four times more likely to receive it, and those who received information about it were six times more likely to take up the vaccine. In an Ethiopian study of female students enrolled in preparatory schools, those who knew a lot about the HPV vaccine had a 2.36-fold higher chance of getting it than those who knew very little [30]. The decision-making process for HPV vaccination among young people is greatly influenced by their level of HPV knowledge [33]. Research shows that young adults and adolescents do not understand human papillomavirus (HPV) and how to prevent it through vaccination [4, 34]. To ensure increased HPV vaccine completion, it is crucial to raise awareness among school nurses and caregivers and enhance information about HPV and its benefits.

Residence plays a significant role in HPV vaccination uptake. In this study, young people living in urban centres were more likely to take up the HPV vaccine. This could be attributed to urban areas having better healthcare access [35], higher awareness of HPV vaccination [35], and community outreach programs [35]. Also, adolescents living in urban centres are exposed to media that provides information on the HPV vaccine and its importance, leading to higher vaccination rates among adolescents. To close the gap in HPV vaccination rates between rural and urban areas, targeted initiatives and better healthcare facility accessibility are required.

Schools play a critical role in promoting HPV vaccination and reducing HPV-related diseases. This study revealed that adolescents who were educated about the HPV vaccine and cervical cancer at school were five times more likely to receive the vaccine. This could be attributed to the fact that schools provide access to many adolescents, are convenient for families, and encourage peer support for vaccination [36]. School-based vaccination programs have positively influenced parents’ attitudes towards HPV vaccination [37]. School nurses play a crucial role in promoting HPV vaccination among children and adolescents by providing education, encouragement, and administration of the vaccine series. Although schools offer a chance to get vaccinated, it could also pose a problem for teenagers living with HIV who need to take more doses than their non-immune-compromised peers. Stigmatisation may result from this. To accommodate immunocompromised teenagers, it is necessary to provide many locations that may be utilised for HPV vaccination. Increasing the rate of HPV vaccination among young people and adolescents who are HIV positive can be achieved through the integration of this vaccine into antiretroviral treatment clinics.

Promoting and providing the HPV vaccine is an important responsibility of healthcare workers. The research found that healthcare professionals’ advice significantly increased the HPV vaccine adoption rate among adolescents by four times. Health workers are crucial in recommending HPV vaccination to patients [38, 39], providing education and support, and addressing misconceptions [38, 39]. Effective patient-provider communication is key for successful vaccination programs and optimal coverage rates [39]. Healthcare workers need to undergo training, education, and community-based outreach programs.

The distance from home to the vaccination location significantly influences HPV vaccine uptake. In this study, we found that adolescents living within a distance of less than 5 km from the health facility were more likely to take the HPV vaccine. These results are similar to studies done; these findings coincide with the findings found in the Kabarole and Lira district in Uganda [40, 41], which found that longer distances associated with poor transport networks are known to hinder individuals from accessing services at health facilities to receive health services. Improving access and uptake can be achieved by bringing immunisation services closer to where girls live, for example, through ART Clinics.

One important factor in determining the rate of HPV uptake is the accessibility of HPV vaccinations. In this study, the availability of HPV vaccines was found to increase the HPV vaccine uptake among adolescents living with HIV by ten times. Research shows that more people will get the HPV vaccine if it is available at the injection location. Having vaccines on hand at the injection location, according to one study in South Michigan, raised uptake from 11% to 16% [35]. Higher HPV vaccine uptake was also linked to outreach immunisation techniques and the availability of sufficient vaccinations, according to a Ugandan study [35]. This is because vaccine availability in convenient places or clinics reduces travel time and costs as it also creates a sense of vaccination importance and is readily accessible, which increases the uptake. There is a need to consider ART clinics, community outreach programs, and school-based initiatives as among the methods that have been proposed to increase vaccine accessibility.

A person’s age is a major factor in whether or not they get an HPV vaccine. The main target age group for HPV vaccination, according to the World Health Organization (WHO), should be 9–14 years old, with an emphasis on vaccinating girls before their sexual activity. In this study, adolescents aged 15- to 19-year-olds were more likely to uptake the vaccine than those above 19 years. This is contrary to some studies that found that older adolescents, particularly those aged 16–18 years, showed higher HPV vaccination uptake compared to younger age groups 14–15 years [42]. This could be explained by the fact that the HPV vaccination is primarily recommended for pre-adolescent and adolescent girls, specifically targeting those aged 9–14 years who are in school, and the immunisation mainly occurs in schools. The rate of HPV vaccine uptake differs with age, according to the studies. For instance, a Ugandan study indicated that compared to females aged 9–14, HPV vaccination uptake was lower among girls aged 15–18 [40, 43]. Addressing these age-related disparities in HPV vaccination will be crucial for improving population-level coverage and cancer prevention.

### Limitations

Firstly, the study’s cross-sectional nature limits its ability to prove a causal link between the identified factors and the uptake or completion of the HPV vaccine. Additionally, it was conducted in a single ART clinic, which might restrict the applicability of the findings to other environments or to HIV-positive adolescents who are not in regular care. Some data were based on self-reports, which could introduce potential recall and social desirability biases. The study primarily examined individual and facility-level factors without considering the health system’s or community’s broader influences. Lastly, the exclusion of males and younger adolescents means that the results cannot be generalised to all young people living with HIV.

## Conclusion

The initiation and completion of HPV vaccination among HIV- positive girls are both crucial for preventing HPV related diseases and cancers. In this study the completion rate is very low. Vaccination against human papillomavirus (HPV) must be prioritised in the HIV-positive youth population. The findings suggest a need for targeted policies to improve vaccine uptake among HIV-positive youth, including better education, healthcare provider engagement, and system support for follow-up care. This will guide targeted interventions in achieving the WHO’s target of 90% immunising all adolescent girls by 2030. 

## Supplementary Information

Below is the link to the electronic supplementary material.

Supplementary Material 1

## Data Availability

The acquired and/or analysed data are not publicly available because of the lack of authorisation from the children’s legal guardians and the agreement with the Research Ethics Committee that the database would remain with the corresponding author only. However, the corresponding author can make all data available upon reasonable request.
