# Supplementary material for: Human papillomavirus (HPV) vaccination uptake (initiation and completion): factors associated with uptake of the human papillomavirus (HPV) vaccine among young people living with HIV in Uganda
Source: BMC Health Serv Res. 2026 Jun 27;26:1269. doi: 10.1186/s12913-026-15035-7 (PMC13576116; doi:10.1186/s12913-026-15035-7)
Supplement: Supplementary file 1 [file 12913_2026_15035_MOESM1_ESM.pdf]

Survey

6.1.1 Sociodemographic characteristics

1. How old are you? ……………………………………

2. What is your religion?

A) Catholic B) Anglican C) Born Again

D) Muslim E) Others

3. What is your tribe?

A) Muganda B) Others

4. With whom are you staying with?

A) Together with parents or one of the parents B) Relative

C) Friend D) other

5. What is your family type?

A) Nuclear family B) Extended family C) I don't know

6. What is your current education status?

A) At school B) out of school

7. What is your level of education?

A) No education B) Primary level

C) Secondary ordinary level D) Tertiary education

8. Where is your residence located?

A) Urban B) Rural

9. How many are you in your household? ……………………………

10. What is the educational level of your caretaker?

A) No education B) Primary education

MAKERERE UNIVERSITY
SCHOOL OF HEALTH SCIENCES
APPROVED
VALID UNTIL
12 DEC 2024
RESEARCH & ETHICS COMMITTEE
P. O. BOX 7072, KAMPALA

C) Secondary ordinary level D) Tertiary education

11. Employment status of caretaker

A) Employed B) Unemployed

6.1.2 Knowledge about HPV and HPV vaccine

12. Human papillomavirus (HPV) vaccine is a vaccine used to prevent Human papillomavirus infection, have you ever heard of it? Have you ever heard of HPV Vaccine?

A) Yes B) NO

If **NO, SKIP to QN… 17**

13. HPV vaccine is used to prevent Human papillomavirus infection and cervical cancer.

A) Yes B) NO C) I don't know

14. What is the recommended number of dose/doses of HPV vaccine for HIV positive adolescent girls?

A) One dose B) Two or more doses C) I don't know

15. What is the ideal time HPV vaccine best recommended?

A) Less than 9 years B) 9-14 years

C) Can be provided at any age D) I don't know

16. Where can you receive the HPV vaccine?

A) Health facility B) community outreach C) School

D) ART clinic E) Other

MAKERERE UNIVERSITY
SCHOOL OF HEALTH SCIENCES
APPROVED
VALID UNTIL
12 DEC 2024
RESEARCH & ETHICS COMMITTEE
P. O. BOX 7072, KAMPALA

6.1.3 Attitudes towards HPV vaccination

Questions		Strongly disagree				
17	It is important for young girls to receive the HPV Vaccine					
18	HPV vaccine is effective in protecting girls against cervical cancer.					
19	Would you accept to receive the HPV vaccine					
20	May your parents/caretaker let you have the HPV vaccine?					
21	May your friends/ peers let you have the HPV vaccine					
22	May your religious leaders/religion let you have the HPV vaccine.					

6.1.4 Information about HPV Vaccine

MAKERERE UNIVERSITY
SCHOOL OF HEALTH SCIENCES
APPROVED
VALID UNTIL
12 DEC 2024
RESEARCH & ETHICS COMMITTEE
P. O. BOX 7072, KAMPALA

23. Have you received any information about HPV Vaccine before?

A) Yes B) NO

If **NO SKIP to QN 25**

24. If **yes**, from whom? Tick all that apply

A) Health workers B) parents/caretakers C) School

D) Mass media E) Friends/peers F) Religious leaders G) Others

25. Have you ever been taught about HPV or cervical cancer at your school?

A) Yes B) NO C) I don't know

26. Availability of awareness creation about cervical cancer or HPV vaccine at school or community

A) Yes B) NO

27. Do you feel you need more information about HPV Vaccination?

A) Yes B) NO

28. If yes, from whom you prefer? (More than one answer is possible)

A) Health workers B) parents/caretakers C) School D) Mass media E) Friends/peers

F) Religious leaders G) Others

6.1.5 Health system factors

29. What is the distance from your home to the health facility?

A) 0-5 KM B) 6-10 KM C) More than 10 KMs D) Not sure

30. Do health providers/workers talk to you about HPV or cervical cancer at the health facility/ART clinic or outreach?

A) Yes B) NO C) I don't know

31. Do health workers provide you with information about the HPV Vaccine at the health facility/ART clinic or outreach?

A) Yes B) NO C) I don't know

32. Do health workers recommend to you or encourage you to receive the HPV vaccine?

A) Yes B) NO C) I don't know

33. Are HPV vaccines available in your nearest Health facilities?

A) Yes B) NO C) I don't know

6.1.6 HPV Vaccine uptake

MAKERERE UNIVERSITY
SCHOOL OF HEALTH SCIENCES
APPROVED
VALID UNTIL
12 DEC 2024
RESEARCH & ETHICS COMMITTEE
P. O. BOX 7072, KAMPALA

34. Have you received the HPV Vaccine?

A) Yes B) NO C) I don't know

If **NO, SKIP to QN….39**

35. If **YES,** how many doses?

A) 1 dose B) 2 doses C) 3 doses

36. Where did you receive the 1st dose from?

A) Health facility B) community outreach C) School

D) ART clinic E) Other

37. For those who received the 2nd dose, where did you receive the second dose of HPV Vaccine from?

A) Health facility B) community outreach C) School

D) ART clinic E) Other

38. For those who received the third dose, where did you receive the 3rd dose of HPV Vaccine from?

A) Health facility B) community outreach C) School

D) ART clinic E) Other

39. What do you think would encourage or motivate you to receive the HPV Vaccine? **Tick all that apply**

1. Availability of enough vaccines
2. Recommendation and counselling from health workers
3. Parental or caretaker support toward HPV vaccination
4. Having information about HPV vaccine i.e., education and awareness campaigns on HPV vaccination
5. Support from friends/ peers for HPV vaccination

MAKERERE UNIVERSITY
SCHOOL OF HEALTH SCIENCES
APPROVED
VALID UNTIL
12 DEC 2024
RESEARCH & ETHICS COMMITTEE
P. O. BOX 7072, KAMPALA

6. HPV vaccination is free of charge
7. Believing in the benefits of HPV vaccination
8. Accessibility to HPV vaccination services
9. Others specify ....

40. What do you think would prevent you from receiving the HPV Vaccine? **Tick all that apply.**

    1. Less confidence in its benefits
    2. Negative attitudes towards the vaccine
    3. Vaccine concerns about safety and effectiveness and fear of side effects
    4. Long distances to vaccination sites
    5. Lack of knowledge and information about the HPV and its vaccine
    6. Unfriendly health workers
    7. Denied by family, friends/ family or peer influence
    8. Cultural or religious beliefs
    9. Inadequate vaccines
    10. Limited access to health facilities

Other specify...

MAKERERE UNIVERSITY
SCHOOL OF HEALTH SCIENCES
APPROVED
VALID
12 DEC 2024
RESEARCH & ETHICS COMMITTEE
P. O. BOX 7072, KAMPALA
